# The Era of the FLips: How Spike Mutations L455F and F456L (and A475V) Are Shaping SARS-CoV-2 Evolution

**DOI:** 10.3390/v16010003

**Published:** 2023-12-19

**Authors:** Daniele Focosi, Pietro Giorgio Spezia, Federico Gueli, Fabrizio Maggi

**Affiliations:** 1 North-Western Tuscany Blood Bank, Pisa University Hospital, 56124 Pisa, Italy; 2 National Institute for Infectious Diseases “Lazzaro Spallanzani” IRCCS, 00149 Rome, Italy; pietro.spezia@inmi.it (P.G.S.); fabrizio.maggi@inmi.it (F.M.); 3 Independent Researcher, 22100 Como, Italy; fedegu@gmail.com

Convergent evolution of the SARS-CoV-2 Spike protein has been mostly driven by immune escape, in particular by escape to the viral infection-neutralizing antibodies (nAbs) elicited by previous infections and/or vaccinations [1]. These immune escaping mutations usually come at a cost for SARS-CoV-2 fitness, often compromising binding to the ACE2 receptor. So, in order to be competitive, a novel SARS-CoV-2 sublineage has to first largely increase the affinity of the Spike protein for the ACE2 receptor, so that it can later accommodate further immune escape mutations. Recently, the S:F456L mutation has been convergently acquired by many independent XBB.1* sublineages and by a single BA.2.75.3-derived sublineage (DV.7.1), soon followed by the S:L455F mutation, a phenomenon referred to as “FLip” (from the initials of the mutated amino acid residues). The acquisition has sometimes been detected as simultaneous (see the lower part of Figure 1), but it should be considered that low genomic surveillance rates in 2023 could account for missing the intermediate step. When the parent lineage also shows the S:K478R mutation—either occurring before the “FLip” (as in JF.1), acquired simultaneously with the “FLip” (as in GW.5), or developed after the “FLip” (as in GK.1.4 and in JR.1.1)—the lineage instead comes under the nickname “FLippeR”. Real world data have shown that lineages which were outcompeted and declining started regrowing in a sustained manner after gaining the FLip, such as XBB.1.5-derived XBB.1.5.70* and JD.1* or CH.1.1-derived DV.7.1. The biological characteristics of one of the FLips, namely HK.3, have recently been reported in detail [2].

Both S:F456L and S:L455F largely increase ACE2 affinity and their combination is synergic [3]. S:L455F also confers some immune escape [4] (and had been observed to emerge after treatment with casirivimab [5]), but FLip lineages remain sensitive to class 1 antibodies [3]. This has also been proven true for BA.2.86 (Pirola) [6], which soon gained fitness in the S:L455S-positive descendant JN.1 [7,8]. Amazingly, JN.1 sequences which have later acquired F456L (nicknamed “Slip’s”) were reported in late November 2023 from France and New Zealand.

Overall, the prevalence of FLip lineages, never seen before since 2019, has grown exponentially in the second half of 2023 [9], and the much-increased serological distance on antigenic chartographies largely explains such expansion [6]. With a plethora of sublineages having acquired the S:F456L mutation (Figure 1), it should be expected that the FLip family is going to grow soon. In particular, more than 70 FLip sublineages, most waiting to be designated, have emerged—especially in China—on the EG.5.1 backbone (tracked in the GitHub issue #537 [10]): its significance and relationship with the immunity built in the general population there after the first two waves [11] should be investigated further.

The impact of different sublineages with FLips on immune escape has been investigated by several laboratories. Using Spike-pseudotyped vesicular stomatitis virus [3] or lentivirus [12,13], XBB.1.5 + S:F456L + S:L455F was found to be 10-fold less sensitive to nAbs than XBB.1.5 + S:F456L in historical BA.5/BF.7 and/or XBB* + S:F486P breakthrough infection sera [3], XBB.1.5 breakthrough infection sera [6], or contemporary sera [13]. The same was true for DV.7.1 [13]. Healthcare workers vaccinated at the third dose with either the monovalent (wild-type) or the wild-type + BA.5 bivalent vaccine similarly had reduced nAb titers against XBB.1.5 + S:F456L + S:L455F than against XBB.1.5 [12]. At the time of writing, no study has been reported yet using live authentic FLip viruses.

With regard to sensitivity to the anti-Spike monoclonal antibodies authorized for clinical use, XBB.1.5 + S:F456L + S:L455F was found to be insensitive to bebtelovimab [3], Evusheld™ [3], and sotrovimab (S-309) [3,12] while preserving sensitivity to SA55 [3], BD56-1854 [3], S3H3 [3], and Omi42 [3].

Another amazing feature of the FLips is the unusually high incidence of second-generation recombinants (XCH, XCL, XCM, XCP, XCR, XCS, XCT, XCY, XCZ, and XDC): while a plethora of non-FLip recombinants have been recently designated (e.g., XCK, XCN, XCQ, XCV, XCU, XCW, XDA, and XDB), this could simply be due to a notoriety bias for the FLips; it remains to be investigated whether they are more prone to recombination [14].

In recent weeks, it has emerged that many of the FLips’ sublineages have further gained the S:A475V mutation, previously seen in a few BA.2.75* descendants (BL.1.5 and BN.1.8). This stepped and ordinate convergence is illustrated in Figure 2. Notably, at the time of writing, FLips + A475V lineages (such as JD.1.1, FL.15.1.1, GW.5.1.1, and GW.5.3.1, as well as many GK.*s) are among the few lineages resisting the fitness of JN.1 in predictive models [15]. Yunlong Cao’s lab recently communicated that S:A475V confers evasion to class 1 antibodies in vitro. A475V has also appeared in the BA.2.86.1 descendant JN.4. Since November 2023 FLips are suffering competition from the fast-growing JN.1* sublineages (Figure 3).

In conclusion, SARS-CoV-2 is again confirming its incredible plasticity in escaping the consolidating human immune response. Since S:F456L, S:L455F, and S:A475V do not occur in the recently marketed XBB.1.5-based “updated” vaccines, the extent to which nAbs in vaccine recipients will provide protection from severe disease remains to be established. Epidemiological monitoring is highly recommended to assess the relationships between specific sublineages and increased clinical severity.

## Figures and Tables

**Figure 1:**
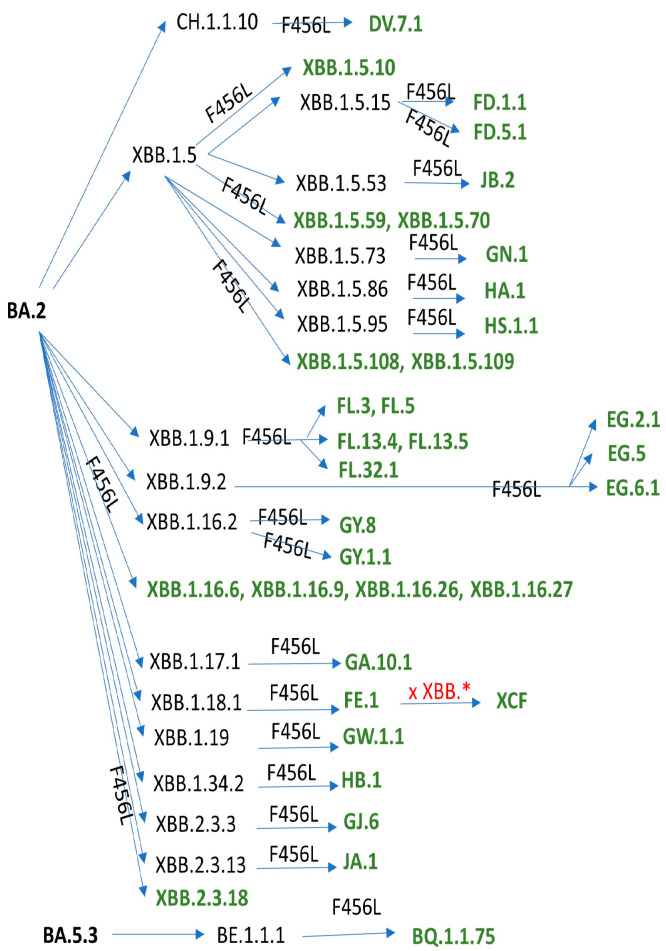
Summary of PANGOLIN-designated SARS-CoV-2 Omicron sublineages which have acquired the S:F456L mutation as of 18 December 2023.

**Figure 2:**
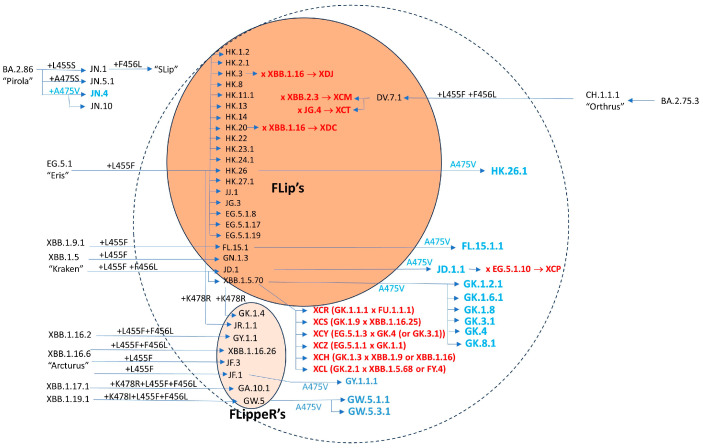
Diagram of SARS-CoV-2 Omicron sublineages which have acquired the FLips and FLIppeR’s mutations so far, with those which have further acquired the S: A475X mutation, as of 18 December 2023.

**Figure 3:**
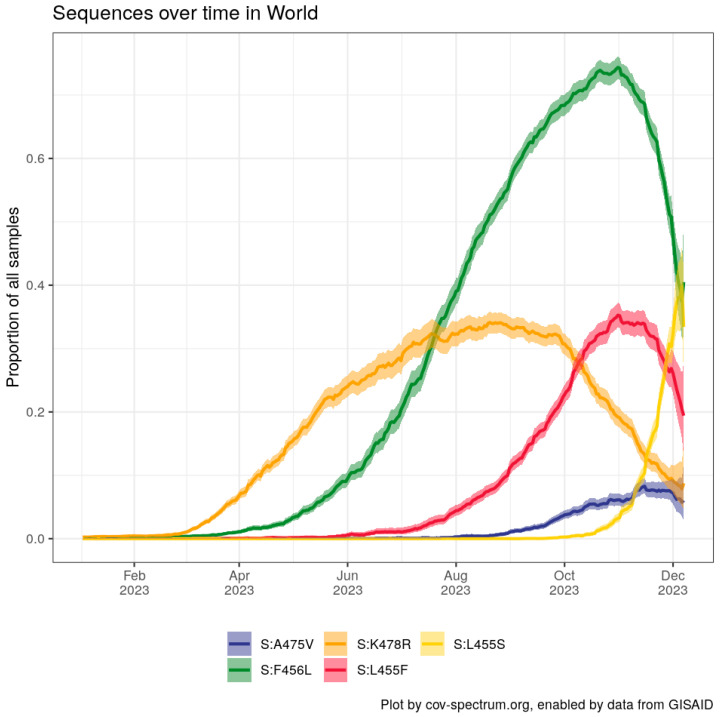
Worldwide prevalence of key Spike protein mutations discussed in the text acquired during 2023. Chart generated with CoV-Spectrum [16]. L455S represents JN.1* sublineages.
